# Passive sensing around the corner using spatial coherence

**DOI:** 10.1038/s41467-018-05985-w

**Published:** 2018-09-07

**Authors:** M. Batarseh, S. Sukhov, Z. Shen, H. Gemar, R. Rezvani, A. Dogariu

**Affiliations:** CREOL, The College of Optics and Photonics, University of Central Florida, Orlando, FL 32816 USA

## Abstract

When direct vision is obstructed, detecting an object usually involves either using mirrors or actively controlling some of the properties of light used for illumination. In our paradigm, we show that a highly scattering wall can transfer certain statistical properties of light, which, in turn, can assist in detecting objects even in non-line-of-sight conditions. We experimentally demonstrate that the transformation of spatial coherence during the reflection of light from a diffusing wall can be used to retrieve geometric information about objects hidden around a corner and assess their location. This sensing approach is completely passive, assumes no control over the source of light, and relies solely on natural broadband illumination.

## Abstract (web summary)

Non-line-of-sight sensing requires purposely controlling some of the properties of light during the measurement process. Here, the authors show that reflection from a diffusive surface preserves some coherence properties and the shape and the distance to an incoherently illuminated object can be measured using the spatial coherence function.

## Introduction

Imaging systems map spatially the distribution of light across an object onto a distant observation plane for further recording and processing. Of course, when objects are too distant or too small to be satisfactorily described by an imaging system, only unresolved sensing is available for estimating physical properties of the object. Whether the object is actively illuminated in a controlled manner, or it is self-luminous, or it is subject to some passive ambient lighting, the imaging procedure is typically constrained by the need for direct view to the object^1^.

**Fig. 1:**
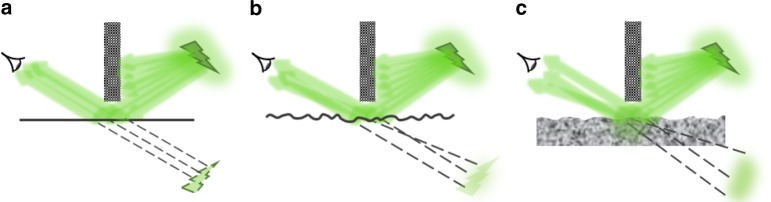
Different non-line-of-sight sensing conditions. **a** A perfect reflector permits imaging around the corner. **b** A broken mirror alters the optical wavefront and impedes forming a clear image. **c** A random medium will alter the reflection even more due to both surface and volume scattering contributions

In non-line-of-sight conditions, an ideal “specular” reflector such as a mirror preserves most of the light properties, including the wavefront, and the imaging procedure is similar to the direct line-of-sight case. Decreasing the mirror’s specularity hinders this capability. A shattered mirror alters the directionality of reflected light and, as a result, only a distorted version of the image can be transferred as illustrated in Fig. 1. The blur can be mitigated if the disturbance can be quantified. Unfortunately, because of the random nature of surface scattering, there are no simple deterministic approaches like ray tracing or conventional diffraction theories to describe the relationship between the incident and reflected optical fields. The situation is further complicated if the light is redirected by a diffusing wall when the interaction is not limited to the surface of the random medium but it extends throughout its volume. In these conditions, recovering the incident wavefront is challenging. The complicated process can be described in terms of the associated transfer matrix, which can be found by controlling the properties of radiation before and after the scattering medium^2–7^.

Nonetheless, some these limitations can be alleviated by an active control of the illumination source. For instance, one can employ time-of-flight approaches to gate the time necessary for light emerging from a controllable source to first reach an object and then a detector capable of discriminating the transient time^8,9^. Imaging angularly small targets hidden around a corner is also possible when using additional measurements performed on reference objects^10^ or when the scene is illuminated with temporally coherent light^11–14^. Sometimes, when an object is diffusively illuminated by a laser and its reflection generates a nonuniform intensity distribution across the scattering wall, detecting the evolution of this intensity allows tracking the object’s movement^15,16^.

Unfortunately, the sensing conditions are significantly more restrictive when one does not have access to the source of illumination. If the object does not generate intensity variations that can be measured, one cannot reconstruct an image in the conventional intensity-based sense^1^. However, even in this rather limiting situation, the object itself acts as the primary (if self-luminous) or the secondary source of partially coherent radiation and relevant information about the object is carried by the statistical properties of the radiated field. The remaining practical question is: do these field properties survive the interaction with scattering obstructions?

In this paper, we demonstrate that spatial correlations of the electromagnetic field can be transferred between the incident and reflected fields in spite of the random nature of interaction with a multiple-scattering medium. Specifically, we show that scattering from randomly inhomogeneous media does not completely destroy the spatial coherence of radiation. This means that a multiple-scattering wall can act as a “broken mirror” for spatial coherence and its distortions can be partially mitigated. We demonstrate that this effect permits retrieving information about the size and shape and allows determining the location of an object even in non-line-of-sight situations.

## Results

### Spatial coherence transfer in reflection off diffusive wall

We consider the situation where radiation from an incoherent source (target) reflects off a scattering surface, e.g. a painted wall, and propagates further until it reaches a detector, which can measure its spatial coherence function (SCF) ${\mathrm{\Gamma }}\left( {{\mathbf{r}},{\mathbf{s}}} \right) = \left\langle {E\left( {{\mathbf{r}} + ({{\mathbf{s}}}/{2}}) \right)E^ \ast \left( {{\mathbf{r}} - ({{\mathbf{s}}}/{2}}) \right)} \right\rangle$. Here, *E*(**r**) is the electric field at the location **r** and **s** is the distance between the points for which the field similarity is being measured (shear).

**Fig. 2:**
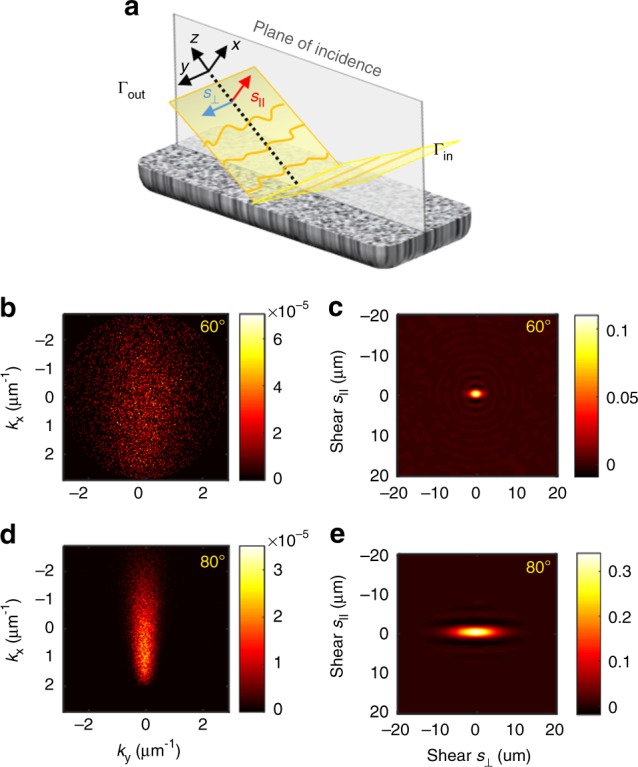
Anisotropic transfer of spatial coherence. **a** Schematic representation of the field reflected from a diffusive wall and its SCF assessed for in-plane *s*_||_ and out-of-plane *s*_⊥_ shears. **b**, **d** Angular distributions of specific intensity corresponding to 60° and 80° angle of incidence, respectively. **c**, **e** Corresponding degrees of the spatial coherence. The incident light is fully coherent spatially and the coherence function of the output is evaluated next to the surface. Parameters of the scattering medium are indicated in the Methods. The mean slope of surface roughness of the simulated medium is *σ* = 0.07 rad

It is well known how coherence evolves in free-space propagation^17^. Thus, certain information about the source can always be extracted by measuring the coherence of the light at distant locations^18^. However, upon reflection from a scattering medium, it is expected that SCF is affected in a way that may complicate this reconstruction procedure. Let us examine the general situation of partially coherent light incident onto a scattering medium as shown in Fig. 2a. Intuitively, one can anticipate that the coherence degrades due to the additional randomization of light and the information about the source of light deteriorates. To mitigate the influence of this interaction, one needs to understand how the coherence properties transform during reflection.

The transformation of SCF in reflection is well understood only for homogeneous, plane−parallel interfaces^19^. Earlier studies also addressed, to a certain degree, the phenomenology of coherence degradation but only in transmission through inhomogeneous media^20,21^. Recently, we developed a Monte Carlo technique that permits estimating the transformation of SCF in multiple-scattering media^22^. This method uses the directions **u** = (**u**_T_, *u*_*z*_) and weights of the “photons” leaving the random medium to evaluate the specific intensity of the scattered field *I*_S_(**r**, **u**) from which the SCF can be evaluated through a Wigner transform^23^:

1 $${\mathrm{\Gamma }}\left( {{\mathbf{r}},{\mathbf{s}}} \right) = {\int} {I_{\mathrm{S}}} \left( {{\mathbf{r}},{\mathbf{u}}} \right)\frac{{{\mathrm{exp}}({\mathrm{i}}k\,{\mathbf{s}}{\mathbf{u}}_{\mathrm{T}})}}{{\left| {u_z} \right|}}{\mathrm{d}}^2u_{\mathrm{T}},$$

where *k* is the wavenumber. The partially coherent beam propagates along the *z*-axis and **u**_T_ is the projection of vector **u** onto a plane perpendicular to *z*. To treat the reflection from realistic scattering media, we augmented this method with a proper description of the surface roughness (see Methods and Supplementary Note 1). Monte Carlo simulations show that light reflected from inhomogeneous media can be effectively described as the superposition of a multiple-scattering component originating in the bulk and the single scattering at the surface (Supplementary Note 1). We found that for typical painted walls the volume scattering randomizes significantly the set of directions **u** corresponding to the incident field and, according to Eq. (1), the coherence information carried by this component is severely altered or even destroyed. However, the inherent single scattering at the surface of any diffusive wall leads to a much smaller randomization of the field, as we will show later.

Energetically, the volume scattering overwhelms the surface one. In the total energy balance, the contribution of surface scattering is only 4% for normal incidence and increases for larger angle of incidence (see Supplementary Note 1). Nevertheless, close to the specular direction, the specific intensity *I*_S_(**r**, **u**) corresponding to surface scattering can be quite high in the case of relatively smooth surfaces as illustrated in Fig. 2b, d. As can be seen, the single scattering contributions lie on top of a much broadly spread background corresponding to the volume scattering but this could be effectively isolated by restricting the angular range of a measurement, i.e. the field of view.

The coherence function is obtained from the specific intensity using Eq. (1) and, as can be seen in Fig. 2c, e, its extent is rather limited spatially. But, most interestingly, the coherence degradation process is not isotropic. We find that, perpendicular to the scattering plane, the spatial coherence Γ(*s*_⊥_) survives much better than for in-plane *s*_|_ shears. In fact, this difference between the two corresponding coherence lengths, $l_{\mathrm c}^ \bot$ and $l_{\mathrm c}^\parallel$, increases with the angle of incidence, which is an effect closely related to the “glitter path” phenomenon: the elongated reflection of a low Sun or Moon on the water’s surface. In this case, the angular spread of wavevectors is determined by the angle of incidence *θ* and the properties of the rough surface^24,25^. From the Wigner transformation in Eq. (1), one can then infer the coherence length $l_{\mathrm c}^ \bot \propto \left( {\sigma \,\,{\mathrm{cos}}\,\,\theta } \right)^{ - 1}$.

**Fig. 3:**
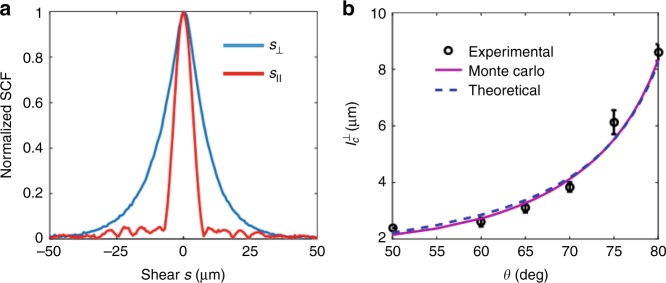
“Glitter path” effect in reflection from random media. **a** Experimental values of the normalized spatial coherence for in-plane *s*_||_ and off-plane *s*_⊥_ shear corresponding to 80° incidence angle. **b** Experimental and simulated values of the off-plane coherence length $l_{\mathrm c}^ \bot$ as a function of the angle of incidence *θ*. Both the source and detection system are located 1 m away from the multiple-scattering wall. The procedure of measurement is detailed in Supplementary Note 3. The solid line represents the Monte Carlo fit to the experimental data from which the average slope of the surface roughness was estimated to be *σ* = 0.07 rad. The dashed line is the corresponding analytical expression $l_{\mathrm c}^ \bot \propto \left( {\sigma \cos \theta } \right)^{ - 1}$. The coherence length (half-width at half-maximum of SCF) of the field incident on the wall is 132 µm. The error bars represent the standard deviation of four independent measurements of coherence length

We analyze this effect in detail using both Monte Carlo simulations and the complex-valued SCF measurements using the Dual Phase Sagnac Interferometer (DuPSaI) procedure detailed in Supplementary Note 3 ^26^. A typical example of measured SCF for reflection from a diffusive wall (estimated transport mean free path 0.9 µm) is presented in Fig. 3a showing a significant difference between in-plane and off-plane shears. Moreover, in Fig. 3b one can clearly see the monotonic behavior of $l_{\mathrm c}^ \bot$ over a significant range of angles of incidence *θ*. The fact that, in certain conditions, the spatial coherence survives in spite of the medium’s diffusiveness can be used to recover information about the source even in non-line-of-sight circumstances.

### Using spatial coherence to estimate the distance to target

An analytical description for the transformation of the complex SCF in reflection was derived in Supplementary Note 2. The coherence function Γ(**r**, **s**; *z*) of the reflected field is essentially the product of the free-space coherence function Γ_0_(**r**, **s**; *z*) propagating the total distance *z* = *z*_1_ + *z*_2_ and an apodizing function Γ_A_(**s**), which depends on both the distance from the object to the wall *z*_1_ and the distance from the wall to the DuPSaI *z*_2_. The phase of the measured complex SCF from reflection coincides with the phase of SCF of a light field propagating in free space over the same distance. For light propagating in free space, the angular position of an incoherent source is encoded in the phase of complex coherence function^27^. The phase of SCF in the observation plane, *ψ* = (*k*/*z*)*s*_⊥_*y*, depends on the total distance *z* to the object, the shearing *s*_⊥_ and the displacement *y* of the detector with respect to the optical axis (as shown in more detail in Supplementary Note 5). Thus, to extract the absolute distance to the source, one can perform measurements of the complex coherence function at several locations and then triangulate to find the object location. The procedure is somewhat similar to the binocular disparity (parallax) concept, i.e. the positional difference between the two projections of a given point in space, and is similar to the way in which the location of nearby stars is determined in astronomy^28^.

**Fig. 4:**
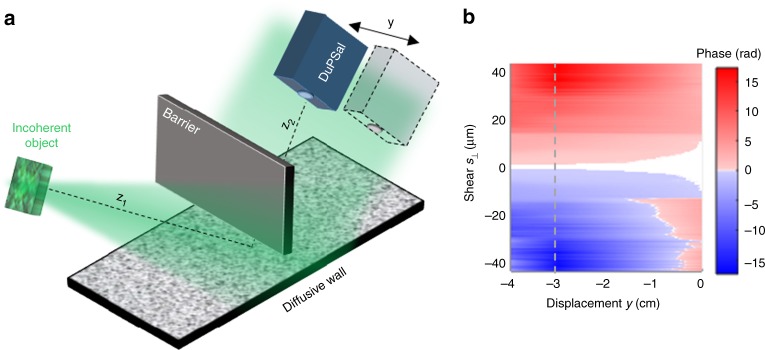
Distance recovery from coherence measurements. **a** The object-to-wall distance is *z*_1_ = 80cm, the angle of incidence is *θ* = 80°, and the complex spatial coherence function of the scattered field is measured at *z*_2_ = 100 cm from the diffusing wall. The coherence detector (DuPSaI) was translated up to 4 cm from the optical axis as indicated. **b** Two-dimensional phase map of the measured coherence function corresponding to different transversal position of DuPSaI, the phase measurement that was used to recover the total distance to the target is represented by the dotted line

As a result, the distance to the object can be obtained from multiple phase measurements of the reflected SCF at different positions *y* as schematically depicted in Fig. 4a. In our demonstration, the incoherent source was created by illuminating a rough object (7.5 cm square) with broadband light emitted from an LED with 30 nm bandwidth and a central wavelength of 525 nm. Light propagated *z*_1_ distance, bounced off a rough scattering wall covered with a thick layer of white paint, and the complex coherence function of the reflected field was measured at a distance *z*_2_ away as shown in Fig. 4a. The phase of SCF was evaluated in the direction *s*_⊥_ that minimizes the coherence degradation. Multiple measurements were performed by displacing the detector up to 4 cm away from the specular direction. The measured phase map is shown in Fig. 4b. By linearly fitting the phase map with the expression *ψ* = (*k*/*z*)*s*_⊥_*y* along shear *s*_⊥_ for a known displacement *y*, the total distance can be recovered. In this example, the SCF phase obtained for a displacement *y* of 3 cm (dotted line in Fig. 4b) and 4 cm was sufficient to recover the 180 cm total distance *z* to the object with a precision better than 2%.

### Using spatial coherence to evaluate target size and shape

The apodization effect of the diffusing wall mentioned before can be numerically evaluated from known properties of the scattering wall or it can be obtained directly by measuring the SCF in conditions similar to the one illustrated in Fig. 3. Of course, this apodizing function should be properly scaled ${\mathrm{\Gamma }}_{\mathrm{A}}\left( {{\boldsymbol{s}};z_1} \right) = {\mathrm{\Gamma }}_{\mathrm{A}}\left( {\alpha {\boldsymbol{s}};z_1^\prime } \right)$ according to the overall distance to the target. The scaling factor $\alpha = ({{z_1}}/({{z_1 + z_2}}))(({{z_1^\prime + z_2}})/{{z_1^\prime }})$can be estimated in advance from the phase measurements as shown before and it depends on the distance *z*_2_ from the wall to the DuPSaI and two different distances *z*_1_ and $z_1^\prime$ between the source and the wall. The entire procedure is detailed in Supplementary Note 4.

By measuring Γ(**r**, **s**), we were able to recover the unperturbed SCF, Γ_0_(**r**, **s**), by dividing the coherence function reflected from the wall by the apodizing function, ${\mathrm{\Gamma }}_0\left( {{\mathbf{r}},{\boldsymbol{s}}} \right) = {\mathrm{\Gamma }}\left( {{\mathbf{r}},{\boldsymbol{s}}} \right){\mathrm{\Gamma }}_{\mathrm{A}}^{ - 1}({\boldsymbol{s}})$. Of course, the quality of this reconstruction depends on both Γ_A_(**s**) and the level of inherent noise in an experiment. Nevertheless, in practical applications the recovery procedure is essentially influenced only by the width of Γ_A_(**s**), which is represented by the extent of $l_{\mathrm c}^ \bot$ shown in Fig. 3b.

**Fig. 5:**
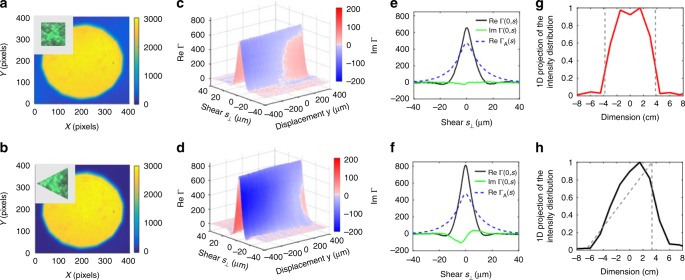
Shape recovery from coherence measurements. **a**, **b** The intensity distribution across the DuPSaI field of view corresponding to the square and equilateral triangle objects, respectively. **c**, **d** Plots of real and imaginary components of SCF measured for the square and equilateral triangle objects, respectively. The imaginary component is color coded and superposed on the 3D representation of the real part of SCF. **e**, **f** Variations of real and imaginary SCF components at *y* = 0. The corresponding apodizing function Γ_A_(*s*) is also indicated by dashed lines. **g**, **h** The 1D projection of the intensity distributions recovered from SCF measurements (solid lines) together with the actual intensity profiles evaluated across the targets (dotted lines)

From this, effectively unperturbed SCF, the one-dimensional projection of the intensity distribution across the target can then be found through a Fourier transformation

2 $$I_s\left( {u_y} \right) = \frac{k}{{2\pi }}{\int} {{\mathrm{\Gamma }}_0} \left( {s_y} \right){\mathrm{exp}}\left( { - {\mathrm{i}}ks_yu_y} \right){\mathrm{d}}s_y$$

as follows from van Cittert−Zernike theorem^29^. In general, the procedure is valid along any direction of shear and the entire intensity distribution across the source could be recovered. The scattering from the diffusing wall however affects the coherence information differently along different directions as shown in Fig. 3. In the following we use the out-of-plane *s*_⊥_ shearing direction where the spatial coherence is least affected. For objects which are uniformly illuminated, the reconstructed intensity distribution provides geometric information about the object and its angular dimension as demonstrated in Fig. 5. Furthermore, using the known distance *z* estimated from the SCF phase, the angular dimensions can be directly converted to absolute values.

Two examples of shape reconstruction are illustrated in Fig. 5. The two targets are equal-area objects, one that is symmetric along the shear direction (square) and one that is not (triangle). We emphasize that in our conditions of operation there are no discernable intensity variations across the field of view as can be seen in Fig. 5a, b. Therefore, in this far-field setting, traditional imaging approaches fail and the targets are unresolved. The complex SCFs, however, are quite different as seen in 5c, d. Notably, the difference in the object symmetry reveals itself in the imaginary parts of the measured SCFs shown in Fig. 5e,f^26^. Moreover, the one-dimensional projection of the intensity distributions along the direction of shear are recovered rather well, which allows to differentiate the shape of the objects as seen in Fig. 5g, h. Within the current field of view of our shearing-based experimental setup, the Pearson coefficient evaluated with respect to the expected intensity profile is 0.93 and 0.89 for the square and triangle, respectively.

## Discussion

Traditional optical imaging requires either straight-line access to the object or a specific arrangement of specular reflectors that create a wrapped version of unobstructed imaging. Non-line-of-sight sensing can also be achieved but only by purposely controlling some of the properties of light during the measurement process. In this Letter, we have shown that information about a non-line-of-sight object can be obtained completely passively without using mirrors and without any access to the source of natural light. For this, we exploit a higher-dimensionality degree of freedom of the optical field. We have shown that the spatial coherence properties of light are not completely destroyed upon reflection from a scattering medium especially for shears perpendicular to the plane of incidence (“glitter path” effect). Moreover, the effect of incoherent volume scattering can be effectively suppressed in practice by limiting the field-of-view of the detection instrument. This proves that, in certain conditions of incidence, a diffuse reflector can act as a “broken mirror” for the complex coherence function of light, which can still permit recovering relevant information about the object.

The recovery procedure was validated using measurements along the out-plane direction where the coherence information is best preserved. Extensions of this method for two-dimensional shape recovery are possible using a plurality of four-dimensional SCF measurements within the available space. Additional information about the scene such as the statistical properties of the illuminating radiation can be recovered from higher-order coherence measurements that go beyond field−field correlations. Finally, in the present demonstration we used an incoherent reflector as our object. However, the approach can be easily extended to absorbing targets by invoking the Babinet’s complementarity principle^30^.

We have considered circumstances when the light, whether produced by the object or originating from another source, reaches the detector only after intermediate scattering from a diffusive wall. This generic setting where the direct vision is impeded is typical for numerous sensing applications ranging from medicine to defense.

## Methods

### Monte Carlo simulations

For the Monte Carlo simulations of volume scattering, we used typical parameters of white paints: TiO_2_ particles with a diameter 200 nm, refractive index 2.6763, and a fractional volume 10% distributed in a matrix with refractive index 1.5. The thickness of the simulated layer is 0.6 mm. We found that the Kirchhoff approximation for the description of surface roughness and a Gaussian distribution of the local slopes^31^ allows both a simple Monte Carlo implementation and a satisfactory description of experimental results. The mean surface slope was determined by matching the outcome of the Monte Carlo simulation to the measured SCF of reflected light for different angles of incidence ranging from 50° to 80°. From the small value of the slope variance (70 mrad) obtained from the fitting one can conclude that for these materials the shadowing effects are insignificant^32^.

### Multiply scattering wall

For the reflection experiments reported here we used a diffusing reflector consisting of a large area drywall painted with commercial white paint (BEHR Premium Plus Ultra Pure White Eggschell Zero VOC interior paint).

### SCF measurements

The complex SCF was measured using a fully automated wavefront shearing interferometer. The instrument combines a Sagnac interferometer integrated with a telescopic imaging system and permits determining the real and imaginary part of the complex SCF from only two measurements, thus the name Dual Phase Sagnac Interferometer (DuPSaI)^26^.

For the coherence measurements, the light source was a high-power LED with bandwidth of 30 nm centered at 525 nm and commercial diffuser (Thorlabs, Solis-525C, 600Grit) with a diameter of 3 mm. The in-plane and off-plane coherence measurements were performed by rotating the Dove prism inside the DuPSaI detector.

The incoherent objects consisted of a rough metallic painted square and an equilateral triangle having the same area of 22.86 cm^2^. The objects were placed at 80 cm from the diffusive wall, which, in turn, was positioned at 1 m from the input aperture of the DuPSaI. The objects were illuminated from the same spatially incoherent source produced by the high-power LED with a diameter of 2 inch and a 600Grit diffuser.

## Electronic supplementary material

Supplementary Information

## Data Availability

The data that support the findings of this study are available from the corresponding author upon reasonable request
