# Supplementary material for: Passive sensing around the corner using spatial coherence
Source: Nat Commun. 2018 Sep 7;9:3629. doi: 10.1038/s41467-018-05985-w (PMC6128873; doi:10.1038/s41467-018-05985-w)
Supplement: Supplementary file 1 — Supplementary Information [file 41467_2018_5985_MOESM1_ESM.pdf]

Supplementary Information:

Passive sensing around the corner using spatial coherence

M. Batarseh, S. Sukhov, Z. Shen, H. Gemar, R. Rezvani, and A. Dogariu

CREOL, The College of Optics and Photonics, University of Central Florida, Orlando, Florida 32816, USA

Supplementary Note 1: Monte Carlo simulations for the reflection of partially coherent light

There are two main components for the simulation of spatial coherence transfer in scattering media: the classical Monte Carlo method for light in scattering media and the Wigner transformation. In the classical Monte Carlo method, incoherent light is described by rays propagating in scattering media with random trajectories. The step size between scattering events and directional change of rays' propagation are determined by physical quantities of scattering media, such as particle size, fractional volume of scatterers, and refractive index contrast. The positions and energy of rays are recorded to reconstruct the intensity distribution both inside and outside of scattering media. However, the classical Monte Carlo method is limited to incoherent light and cannot describe the transfer of spatial coherence.

To generalize the classical Monte Carlo method for the modeling of propagation and transformation of spatial coherence, the Wigner transformation is introduced [1]. The Wigner transformation provides a perspective into the interpretation of spatial coherence in a radiometric fashion.

$$\Gamma(\mathbf{r},\ \boldsymbol{\rho}) = \int I_s(\mathbf{r},\mathbf{u})\frac{\exp(ik\mathbf{s}_\mathrm{T}\mathbf{u}_\mathrm{T})}{|u_z|}d^2\mathbf{u}_\mathrm{T}$$

$$I_s(\mathbf{r},\mathbf{u}) = \left(\frac{k}{2\pi}\right)^2 |u_z| \int\Gamma(\mathbf{r},\boldsymbol{\rho})\exp(-ik\mathbf{s}_\mathrm{T}\mathbf{u}_\mathrm{T})\, d^2\mathbf{s}_\mathrm{T} \quad (1)$$

Where Γ is the spatial coherence function (SCF), I_s is the generalized radiance (specific intensity), $\mathbf{r}$ is the center position, $\mathbf{s}$ is shear, $\mathbf{u}$ is the direction of ray propagation, and k is the wavenumber of light. As shown in the Fourier kernel, the shear $\mathbf{s}$ and the direction of generalized radiance $\mathbf{u}$ are a pair of conjugate variables. In other words, a less spatially coherent source has a more diverse distribution of generalized radiance. The distribution of generalized radiance can be calculated using the positions, directions, and energy of the rays available in the Monte Carlo simulation. Based on the distribution of generalized radiance, the local spatial coherence function can be recovered by a Wigner transformation. In other words, the classical Monte Carlo method can be upgraded to simulate spatial coherence propagation by exploiting the directional information of rays in the scattering media.

For the reflection regime, spatial coherence is affected by both the scattering media (volume scattering) and rough surface (surface scattering). We made three assumptions to simplify the interaction between light and a rough surface [2]. First, when the surface is locally smooth, a wave can be described using a ray (tangent plane approximation). Second, the surface profile is described statistically (statistical approach sufficiency). Third, surface self-shadowing and multiple scattering are neglected. Thus, rough surface is simplified to a Gaussian statistical slope distribution. This statistical slope randomizes the directions of incoming rays; thus, the distribution of generalized radiance becomes more diverse, and the spatial coherence function is narrowed. To summarize simply, surface scattering can also be interpreted in a statistical ray manner, which is consistent with Monte Carlo simulation for volume scattering.

Although natural surfaces should not necessarily satisfy all our assumptions, we found that the conclusions of Monte Carlo simulations describe very well the experimental situations described in the main text.

Monte Carlo simulations for the transformation of spatial coherence in reflection were performed for a scattering medium corresponding to a typically painted surface. Typical parameters of white paints (TiO_2) were used: inclusion particles diameter of 200 nm, the refractive index of particles is 2.67, the

refractive index of the matrix is 1.5, the fractional volume of inclusions is 10%, the surface mean slope is 70 mrad (this value was estimated from the fitting to experimental data, see Fig.3b of the main text), and the thickness of simulated slab is 0.6 mm.

The Monte Carlo simulations uncovered several features of scattering. First, even though the volume scattering happens in a thin subsurface layer (0.6 mm), the corresponding average number of scattering events is rather high (approximately 500 scattering events on average for chosen set of parameters). This leads to a complete randomization of volume scattered light with rays of reflected light uniformly distributed along all directions, as shown in Supplementary Figure 1. Thus, randomized volume scattering does not carry any useful directional information and leads to the light completely losing its coherence properties.

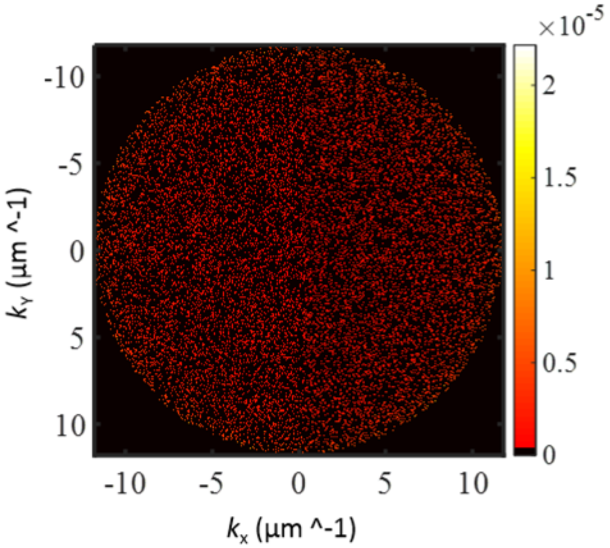

Supplementary Figure 1 | Distribution of generalized radiance for volume scattering.

Second, the randomization of the light scattered from the surface is determined by the mean slope. In general, the randomization of wave vectors occurs differently for the components in and normal to the incidence plane. With the increase of the incident angle, the randomization of the component normal to the plane of incidence decreases (see Figures 2,3 of the main text). Hence, for grazing angles the spatial coherence for shears normal to the plane of incidence survives much better than for shears in the plane of incidence.

Third, energetically speaking, volume scattering usually dominates the surface scattering for incidence angles close to the normal (Supplementary Figure 2). For a larger incident angle, the ratio of surface scattering over volume scattering increases. In other words, the informationally deprived 'noise' from volume scattering decreases as the incident angle increases. It is more practical to view the comparison between surface and volume scattering using the energetic density in the wavevector space. The volume scattering is more or less uniformly distributed over 2π solid angle (Supplementary Figure 1). Surface scattering is distributed over much smaller angular range and concentrated along specular direction of reflection (Figure 2 of the main text). Therefore, the energetic density in wavevector space is dominated by surface scattering when observing in the direction of specular reflection.

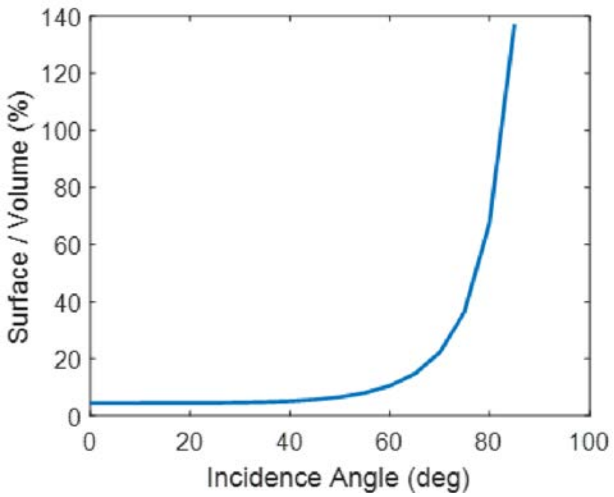

Supplementary Figure 2 | Monte Carlo simulation for energy ratio between surface scattering and volume scattering.

The contribution of volume scattering can be suppressed even more if the field-of-view (FOV) of the detection device is limited to the angles occupied by wave vectors carrying useful information (Supplementary Figure 3). The finite size FOV effectively truncates the significant part of volume scattering, which causes surface scattering to dominate (Supplementary Figure 3.b). Thus, the reflection from a highly scattering medium can be reduced to the reflection from a rough surface – broken mirror.

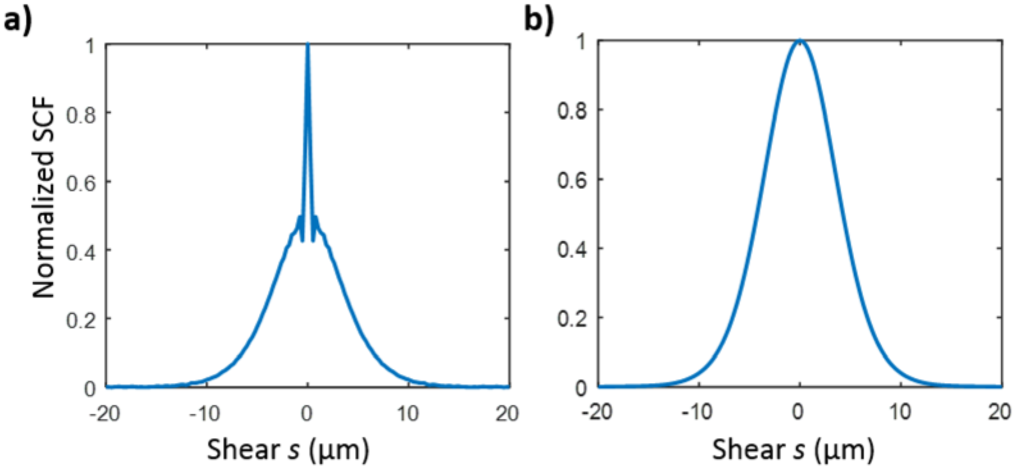

Supplementary Figure 3 | Spatial coherence function measured for different field of view. (a) SCF for the large field of view observation; (b) SCF for a restricted FOV.

Supplementary Note 2: Analytical results for SCF of the light reflected by a rough surface

Monte Carlo simulations allow for a detailed description of transformation of spatial coherence function during reflection. However, to learn about the laws governing this transformation, an analytical description is desired. In attempt to do that, we consider the following problem. The light from incoherent source propagates distance z_1 and then reflects from a rough surface. One needs to determine the coherence of the reflected light after it propagates distance z_2 after reflection.

To simplify the problem, we make the following assumptions:

1) The size of the object is much smaller than a distance to the surface $a \ll z_1$ so, paraxial approximation can be used;
2) The volume scattering can be neglected (the result confirmed by Monte Carlo simulations);
3) The surface is smooth (mean slope is much less than 1) with characteristic dimensions of roughness much larger than a wavelength. In this case, geometric optics approximation of reflection can be used.

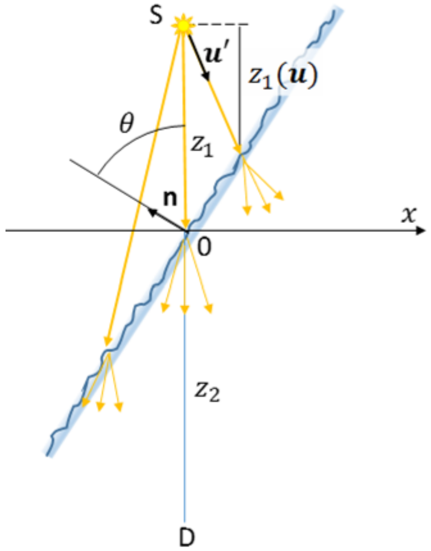

Supplementary Figure 4 | 'Unwrapped' geometry of surface reflection.

First, we consider the case of a point-like source located at the position z_1 (Supplementary Figure 4). The radiation field created by a point source is spatially fully coherent with each point of the wavefront having deterministic wavevector. For a directional vector $\boldsymbol{u}'$, the intersection of the vector with a surface in paraxial approximation is determined by the following expressions:

$$x = \frac{z_1 u'_x}{1 + u'_x \tan\theta},$$

$$y = \frac{z_1 u'_y}{1 + u'_x \tan\theta},$$

$$z_1(\mathbf{u}') = \frac{z_1}{1+u'_x \tan\theta}. \quad (2)$$

Here θ is the angle of incidence. The incident spatially coherent light from a point source is perturbed by a rough surface and becomes partially coherent after reflection. We assume that every original propagation vector $\mathbf{u}'(\mathbf{r}_\perp)$ ($\mathbf{r}_\perp = (x, y)$) is being distributed into a range of directions described by a Gaussian probability function. From prior Monte Carlo simulations and from experiments, we know that perturbation to the light is different in the plane of incidence (along x direction) and perpendicular to this plane (off-plane along y direction):

$$P(\mathbf{u}, \mathbf{u}') = \frac{1}{2\pi\sigma_x\sigma_y} \exp\left[-\frac{(u_x - u'_x)^2}{2\sigma_x^2}\right] \exp\left[-\frac{(u_y - u'_y)^2}{2\sigma_y^2}\right]. \quad (3)$$

Here $P(\mathbf{u}, \mathbf{u}')$ determines the probability distribution of directions $\mathbf{u}$ of scattered photons for a local wave incident along direction $\mathbf{u}'$. As a result of the proposed procedure, for every point on a surface we know the distribution of outgoing wavevectors, which is exactly the definition of specific intensity I_s.

Thus, I_s for the reflected light next to the surface can be found from Eq.(3) by expressing $\mathbf{u}'$ through coordinates $\mathbf{r}_\perp$:

$$I_s(\mathbf{r}_\perp, \mathbf{u}) = \frac{I_0(\boldsymbol{r}_\perp)}{2\pi\sigma_x\sigma_y} \exp\left[-\frac{(u_x - x/(z_1 - x\tan\theta))^2}{2\sigma_x^2}\right] \exp\left[-\frac{(u_y - y/(z_1 - x\tan\theta))^2}{2\sigma_y^2}\right]. \tag{4}$$

Here $I_0(\mathbf{r}_\perp)$ is the intensity of incident wave at the location $\mathbf{r}_\perp$:

$$I_0(\mathbf{r}_\perp) = \frac{I_0\delta s}{(z_1 - x\tan\theta)^{\ 2}}, \tag{5}$$

I_0 is the intensity of the point source and δs is an elemental surface of the point source. The propagation of I_s (in paraxial approximation) is described by a simple relation [3]:

$$I_s(\mathbf{r}_\perp, \mathbf{u}; z_2) = I_s(\mathbf{r}_\perp - z_2(\mathbf{u})\mathbf{u}, \mathbf{u}). \tag{6}$$

Notice that $\boldsymbol{r}_\perp$ in the expression for $I_s(\mathbf{r}_\perp, \mathbf{u}; z_2)$ corresponds to the transversal coordinate at the detection plane $z = z_2$. After straightforward calculations, one obtains

$$z_2(\mathbf{u}) = \frac{z_2}{1 - u_x\tan\theta}. \tag{7}$$

To obtain specific intensity at distance z_2, we perform the following substitutions in Eq.(4):

$$x \to x - \frac{z_2 u_x}{1 - u_x\tan\theta}$$

$$y \to y - \frac{z_2 u_y}{1 - u_x\tan\theta} \tag{8}$$

where x and y are now coordinates in the detection plane. The expression for specific intensity at the observation plane reads

$$I_s(\mathbf{r}_\perp, \mathbf{u}; z_2) = \frac{I_0(\mathbf{r}_\perp)}{2\pi\sigma_x\sigma_y} \exp\left[-\frac{1}{2\sigma_x^2}\left(u_x - \frac{x - u_x z_2 - u_x x\tan\theta}{z_1 + \tan\theta\,(-x + u_x(z_2 - z_1) + u_x x\tan\theta)}\right)^2\right] \times$$

$$\times \exp\left[-\frac{1}{2\sigma_y^2}\left(u_y - \frac{y - u_y z_2 - u_x y\tan\theta}{z_1 + \tan\theta\,(-x + u_x(z_2 - z_1) + u_x x\tan\theta)}\right)^2\right]. \tag{9}$$

$I_0(\mathbf{r}_\perp)$ is now written as

$$I_0(\mathbf{r}_\perp) = I_0\delta s\left(z_1 - x\tan\theta + \frac{z_2 u_x\tan\theta}{1 - u_x\tan\theta}\right)^{-2}. \tag{10}$$

The expressions inside exponential terms of Eq.(9) have very cumbersome form. Assuming paraxial approximation, we can simplify these expressions by leaving only linear terms with respect to u_x, u_y:

$$I_s(\mathbf{r}_\perp, \mathbf{u}; z_2) = \frac{I_0(\mathbf{r}_\perp)}{2\pi\sigma_x\sigma_y} \exp\left[-\frac{1}{2\sigma_x^2}\left(u_x\left(1 + \frac{z_2}{z_1}\right) - \frac{x}{z_1}\right)^2\right]$$

$$\times \exp\left[-\frac{1}{2\sigma_y^2}\left(u_y\left(1 + \frac{z_2}{z_1}\right) - \frac{y\left(1 - (\frac{z_2}{z_1})u_x\tan\theta\right)}{z_1}\right)^2\right]. \tag{11}$$

In Eq.(11), we also assumed that $x\tan\theta \ll z_1$ corresponding to small displacements from optical axis, which was the case for all experimental conditions described in the main text. The spatial coherence function Γ can be found from the Wigner distribution function by a Fourier transform [4]:

$$\Gamma(\mathbf{r}_\perp, s_y; z_2) = \int I_s(\mathbf{r}_\perp, \mathbf{u}; z_2) e^{iks_y u_y} d\mathbf{u}. \tag{12}$$

Here, again, paraxial approximation was assumed. Variable s_y in Eq.(12) denotes the distance between two points with respect to which coherence is measured. When calculating integral (12), one can neglect the spatial variation of intensity (10). The final integral looks like the following:

$$\Gamma(y, s_y; z_2) = \frac{I_0 \delta s}{(z_1 + z_2)^2} \exp\left[-\frac{1}{2}\left(\frac{k s_y z_1 \sigma_y}{z_1 + z_2}\right)^2\right] \exp\left[\frac{i k s_y y}{z_1 + z_2}\right] \exp\left[-\frac{1}{2}\left(\frac{k s_y y z_2 \sigma_x \tan\theta}{(z_1 + z_2)^2}\right)^2\right] \times$$
$$\times \exp\left(-\frac{i x k s_y y z_2 \tan\theta}{z_1 (z_1 + z_2)^2}\right) \quad (13)$$

Eq.(13) represents the coherence function for the field emitted by a point source and reflected by rough surface. One can see that the coherence function $\Gamma(y, s_y; z_2)$ is defined by a product of the expression for a free-space propagating field

$$\Gamma_0(y, s_y; z_2) = \frac{I_0 \delta s}{(z_1 + z_2)^2} \exp\left[\frac{i k s_y y}{z_1 + z_2}\right] \quad (14)$$

and some apodizing factors. For the parameters used in experiment, the influence of last two terms in Eq.(13) is negligible. One of the consequences of Eq.(13) is the decrease the coherence length with an increase of the distance z_1. Surprisingly, this is opposite to what can be observed in a free space propagation of partially coherent light.

Having established the expression for the coherence of a point source, now we can proceed to the case of a finite size object. According to generalized Babinet principle for the coherence function [5], the coherence function for a finite size object can be obtained by summing all the point sources composing this object:

$$\Gamma_{total}(\mathbf{r}_\perp, s_y; z) = \frac{1}{S} \int_S \Gamma(\mathbf{r}_\perp - \mathbf{r}_\perp', s_y; z) d\mathbf{r}_\perp' , \quad (15)$$

where integration is performed over the surface of the object. For nearly flat objects, $z_1 \approx \mathrm{const}$ for all the points composing the object. The final expression for SCF of the light produced by incoherent source and reflected from rough surface has the following form:

$$\Gamma_{\mathrm{total}}(y, s_y; z_2) = \exp\left[-\frac{1}{2}\left(\frac{k s_y z_1 \sigma_y}{z_1 + z_2}\right)^2\right] \Gamma_{\mathrm{total}}^0(y, s_y; z_1 + z_2), \quad (16)$$

where $\Gamma_{\mathrm{total}}^0(y, s_y; z)$ is the spatial coherence function after a free-space propagation over distance z. Similar result for the normal incidence was obtained by [6]. Here this result is obtained in a more elegant way.

Supplementary Note 3: Interferometric measurement of complex SCF

To measure complex spatial coherence function, we built an interferometric wavefront lateral shearing instrument [7]. Two identical copies of partially coherent field are counter propagating through a common path with a phase difference between them created by half-wave and quarter-wave plates (Supplementary Figure 5). To obtain the complex SCF

$$\Gamma(\mathbf{r},\mathbf{s}) = \langle E\left(\mathbf{r}+\frac{\mathbf{s}}{2}\right)E^*\left(\mathbf{r}-\frac{\mathbf{s}}{2}\right)\rangle, \tag{17}$$

we shear these copies by displacing the mirrors along the common path. This allows us to measure the intensity change as a function of the shear of the fields. When both plates are aligned with the fast axis of the polarizer, these two copies have a zero phase difference and the detected intensity can be written as:

$$I^0(\mathbf{r},\mathbf{s}) = I\left(\mathbf{r}+\frac{\mathbf{s}}{2}\right) + I\left(\mathbf{r}-\frac{\mathbf{s}}{2}\right) + 2\mathrm{Re}[\Gamma(\mathbf{r},\mathbf{s})]. \tag{18}$$

Here $I(\boldsymbol{r})$ are the intensities in each of the arms of interferometer. We then rotate the quarter-wave plate by 45-degrees; this introduces a 90-degree phase difference between the two copies of the field and the detected intensity becomes:

$$I^{\frac{\pi}{4}}(\mathbf{r},\mathbf{s}) = I\left(\mathbf{r}+\frac{\mathbf{s}}{2}\right) + I\left(\mathbf{r}-\frac{\mathbf{s}}{2}\right) + 2\mathrm{Im}[\Gamma(\mathbf{r},\mathbf{s})]. \tag{19}$$

At zero shear with the wave plates aligned $I^0(\mathbf{r},0) = 4\cdot I(\mathbf{r})$. This allows us to simply calculate the real and imaginary components of the complex SCF:

$$\mathrm{Re}[\Gamma(\mathbf{r},\mathbf{s})] = 0.5\left(I^0(\mathbf{r},\mathbf{s}) - 0.5\, I^0(\mathbf{r},0)\right)$$

$$\mathrm{Im}[\Gamma(\mathbf{r},\mathbf{s})] = 0.5(I^{\frac{\pi}{4}}(\mathbf{r},\mathbf{s}) - 0.5\, I^0(\mathbf{r},0)) \tag{20}$$

Using two phase delays for SCF measurement gave the name to our device: Dual Phase Sagnac Interferometer (DuPSaI). The introduction of a Dove prism (DP) into our system allows us to measure the SCF along any direction $\mathbf{s}$.

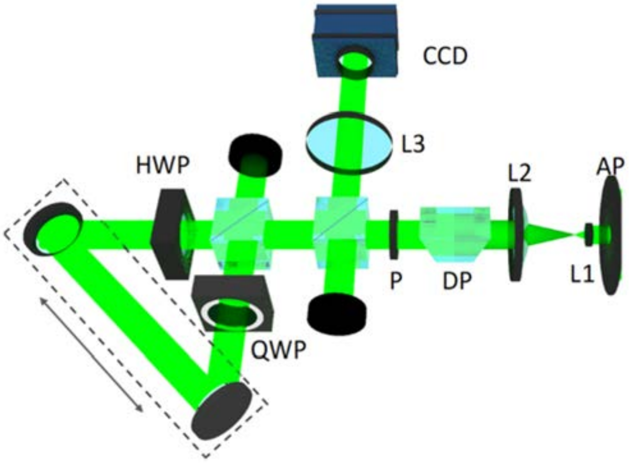

Supplementary Figure 5 | Schematic of the experimental setup (DuPSaI) for measuring the SCF at the plane of the input aperture AP. L1, L2, L3, lenses; HWP, half-wave plate; QWP, quarter-wave plate; DP, Dove prism; P, polarizer.

Supplementary Note 4: Measuring apodizing functions

To reconstruct free-space SCF from experimentally measured surface reflected field, one needs to know the corresponding apodizing function, Eq.(16). This function corresponds to the magnitude of SCF of a point source measured at optical axis $y = 0$, Eq.(13).

To verify experimentally the effect of the distance z_1 on the shape of the apodizing function, we used a quasi-point LED source (3mm in diameter point source) that illuminates the scattering wall from different distances as shown in Supplementary Figure 6.a. When the distance to the wall increases, the magnitude of the coherence function decreases (Supplementary Figure 6.b), a trend that follows our theoretical prediction (16). The experimental apodizing functions have the same shapes and scale down as the distance to the wall z_1 increases. The noticeable difference between theory and experiment is the non-Gaussian shape of the apodizing function that is possibly determined by a non-Gaussian distribution of the surface roughness.

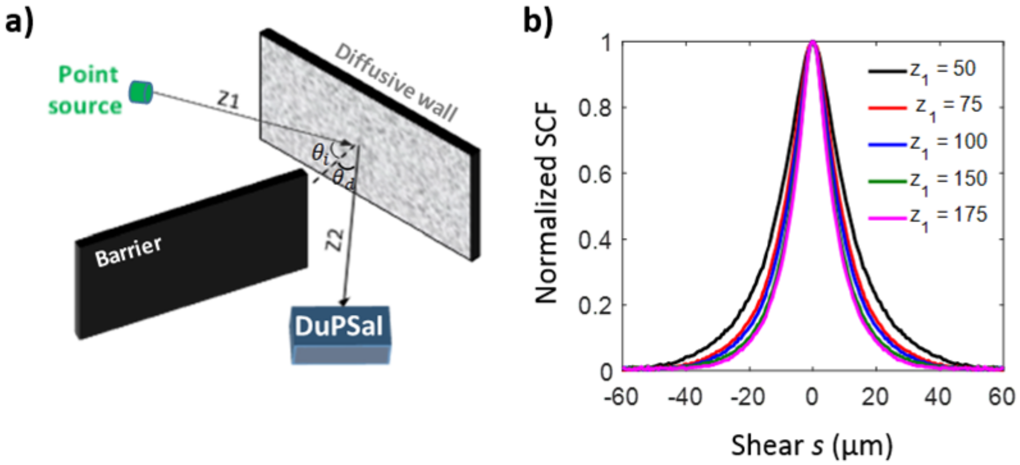

Supplementary Figure 6| Measurement of coherence function for a point source. (a) Reflection geometry: point source-to-wall distance z_1, detector-to-wall distance z_2=100 cm, angle of incidence and detection $\theta_i = \theta_d = 80°$, the point source is 3mm in diameter. (b) Magnitude of the normalized coherence function for point source at different distance from the wall z_1.

Based on the analytical description, the apodizing functions have the same shape but they scale according to the following equation:

$$\Gamma_A(s; z_1) = \Gamma_A(\alpha s; z_1'),$$
$$\alpha = \frac{z_1}{z_1+z_2}\frac{z_1'+z_2}{z_1'}, \quad (21)$$

where z_2 is the distance from the wall to the DuPSaI, z_1 and z_1' are two different distance of the source to the wall. We found that the experimental data follow a similar scaling trend with rather small deviations from the theoretical prediction as seen in Supplementary Figure 7. In practice, a proper apodizing function can be selected from measured values of Γ_A.

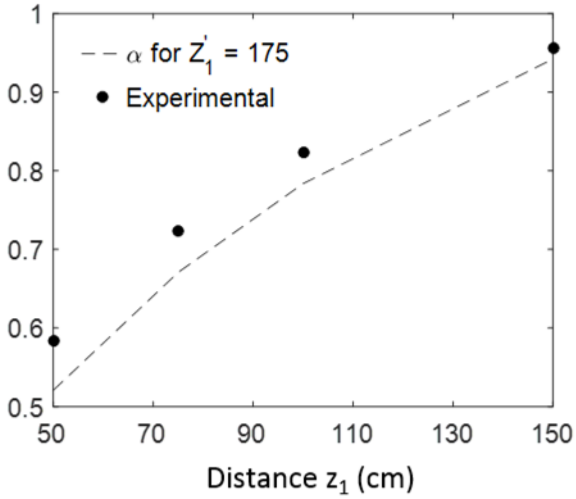

Supplementary Figure 7| Scaling of coefficient α. Apodizing functions are measured at several distances z_1 and scaled to the distance $z_1' = 175$cm. The dashed line is the analytical result.

Supplementary Note 5: Distance to object recovery from SCF measurement

The coherence of the light reflected by a rough surface is determined by Eq.(16). One can conclude from this expression that the phase of this spatial coherence function coincides with the phase of SCF of free space propagation. Thus, we can use the phase of measured coherence function to infer the distance to the source.

As usual, we assume that the dimensions of the source are much smaller than the distance between the source and detector (paraxial approximation). The phase of the coherence function for a free space propagation in this case is determined by the Van Cittert-Zernike theorem [8]:

$$\psi = \frac{\pi}{\lambda z}(r_2^2 - r_1^2) = \frac{2\pi}{\lambda z}(s_x x + s_y y), \quad (22)$$

$\mathbf{r}_i = (x_i, y_i), i = 1{,}2$ represent the distance at observation plane from the optical axis, $s_x = x_2 - x_1$, $s_y = y_2 - y_1$ are shears in x and y directions respectively, $x = (x_2 + x_1)/2$, $y = (y_2 + y_1)/2$ are average points. For shears along y-direction, we have the following equation

$$\psi(s_y, y) = \frac{2\pi s_y y}{\lambda z}. \quad (23)$$

Constructing a 2D map of $\psi(s_y, y)$, distance to the object can be determined by fitting 2D map to the above equation.

Supplementary Note 6: Restoration of 1D intensity distribution from SCF measurements

According to Eq.(16), the free-space spatial coherence function $\Gamma_0(s)$ can be obtained from the experimentally measured SCF $\Gamma(s)$ by dividing it with the apodizing function Γ_A. The corresponding specific intensity can be obtained from $\Gamma_0(s)$ by a Fourier transformation:

$$I_s(u_y) = \frac{k}{2\pi} \int \Gamma_0(s_y) \exp(-iks_y u_y)\, ds_y. \quad (24)$$

The recovered specific intensity represents the angular distribution of intensity across the object, i.e. its nominal shape. To obtain linear dimension of the object, one needs to multiply the obtained angular distribution $I_s(u_y)$ by the previously found distance to the object z.

Supplementary References:

1 Wolf, E. Coherence and radiometry. *JOSA* **68**, 6-17 (1978).
2 Sun, Y. Statistical ray method for deriving reflection models of rough surfaces. *JOSA A* **24**, 724-744 (2007).
3 Gradoni, G., Creagh, S. C., Tanner, G., Smartt, C. & Thomas, D. W. A phase-space approach for propagating field–field correlation functions. *New Journal of Physics* **17**, 093027 (2015).
4 Goodman, J. Introduction to Fourier optics. (2008).
5 Sukhov, S. *et al.* Babinet's principle for mutual intensity. *Optics letters* **42**, 3980-3983 (2017).
6 Nagata, K.-i., Yoshida, N. & Nishiwaki, J. Ensemble-averaged coherence function of light reflected from rough surface: determination of its correlation length. *Japanese Journal of Applied Physics* **9**, 505 (1970).
7 Naraghi, R. R. *et al.* Wide-field interferometric measurement of a nonstationary complex coherence function. *Optics letters* **42**, 4929-4932 (2017).
8 Goodman, J. W. *Statistical optics*. (John Wiley & Sons, 2015).
